# (*E*)-*N*′-[4-(Dimethyl­amino)­benzyl­idene]-2-meth­oxy­benzohydrazide

**DOI:** 10.1107/S1600536810023937

**Published:** 2010-06-26

**Authors:** Shi-Yong Liu, Xiaoling Wang

**Affiliations:** a College of Chemistry & Pharmacy, Taizhou University, Taizhou Zhejiang 317000, People’s Republic of China; b Department of Chemistry, Liaoning Normal University, Dalian 116029, People’s Republic of China

## Abstract

In the title compound, C_17_H_19_N_3_O_2_, the two benzene rings form a dihedral angle of 89.2 (2)°. In the crystal structure, mol­ecules are linked through N—H⋯O hydrogen bonds, forming *C*(4) chains running along the *c* axis.

## Related literature

For the medicinal applications of hydrazone compounds, see: Hillmer *et al.* (2010 ▶); Zhu *et al.* (2009 ▶); Jimenez-Pulido *et al.* (2008 ▶); Raj *et al.* (2007 ▶); Zhong *et al.* (2007 ▶). For hydrazones we have reported previously, see: Liu & You (2010*a* ▶,*b* ▶,*c* ▶). For the crystal structures of similar hydrazone compounds, see: Khaledi *et al.* (2009 ▶); Warad *et al.* (2009 ▶); Back *et al.* (2009 ▶); Vijayakumar *et al.* (2009 ▶). For other related structures, see: Cao (2009 ▶); Xu *et al.* (2009 ▶); Shafiq *et al.* (2009 ▶).
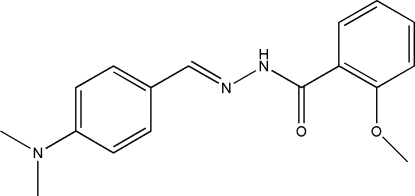


## Experimental

### 

#### Crystal data

C_17_H_19_N_3_O_2_
*M* *_r_* = 297.35
Orthorhombic, 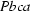

*a* = 24.726 (4) Å
*b* = 15.385 (3) Å
*c* = 8.2700 (15) Å
*V* = 3146.0 (10) Å^3^
*Z* = 8
Mo *K*α radiation
μ = 0.08 mm^−1^
*T* = 298 K
0.20 × 0.17 × 0.13 mm

#### Data collection

Bruker SMART CCD area-detector diffractometer
Absorption correction: multi-scan (*SADABS*; Bruker, 2001 ▶) *T*_min_ = 0.983, *T*_max_ = 0.989
12277 measured reflections
2096 independent reflections
1545 reflections with *I* > 2σ(*I*)
*R*_int_ = 0.045
θ_max_ = 22.7°

#### Refinement

*R*[*F* ^2^ > 2σ(*F* ^2^)] = 0.039
*wR*(*F* ^2^) = 0.106
*S* = 1.04
2096 reflections
202 parameters
H-atom parameters constrained
Δρ_max_ = 0.11 e Å^−3^
Δρ_min_ = −0.15 e Å^−3^

### 

Data collection: *SMART* (Bruker, 2007 ▶); cell refinement: *SAINT* (Bruker, 2007 ▶); data reduction: *SAINT*; program(s) used to solve structure: *SHELXTL* (Sheldrick, 2008 ▶); program(s) used to refine structure: *SHELXTL*; molecular graphics: *SHELXTL*; software used to prepare material for publication: *SHELXTL*.

## Supplementary Material

Crystal structure: contains datablocks global, I. DOI: 10.1107/S1600536810023937/sj5024sup1.cif

Structure factors: contains datablocks I. DOI: 10.1107/S1600536810023937/sj5024Isup2.hkl

Additional supplementary materials:  crystallographic information; 3D view; checkCIF report

## Figures and Tables

**Table 1:** Hydrogen-bond geometry (Å, °)

*D*—H⋯*A*	*D*—H	H⋯*A*	*D*⋯*A*	*D*—H⋯*A*
N1—H1⋯O2^i^	0.90	2.03	2.914 (2)	165

Symmetry code: (i) 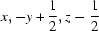.

**Fig. 1.**
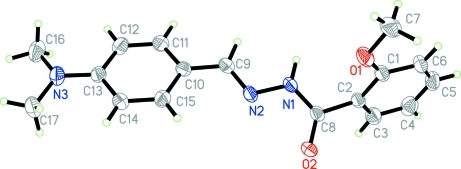
The molecular structure of the title compound. Displacement ellipsoids are drawn at the 30% probability level. Hydrogen atoms are shown as spheres of arbitrary radius.

**Fig. 2.**
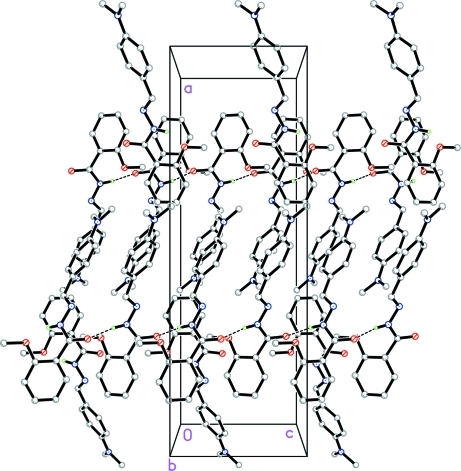
The molecular packing of the title compound, viewed along the b axis. Hydrogen bonds are shown as dashed lines. Hydrogen atoms not involved in hydrogen bonding have been omitted.

## supplementary crystallographic information

### Comment

Considerable attention has been focused on hydrazones and their medicinal applications (Hillmer *et al.*, 2010; Zhu *et al.*, 2009; Jimenez-Pulido *et al.*, 2008; Raj *et al.*, 2007; Zhong *et al.*, 2007). The study on the crystal structures of such compounds is of particular interest (Khaledi *et al.*, 2009; Warad *et al.*, 2009; Back *et al.*, 2009; Vijayakumar *et al.*, 2009). As a continuation of our work on such compounds (Liu & You, 2010*a*,b,c), we report herein the crystal structure of the title new hydrazone.

The molecular structure of the title compound is shown in Fig. 1. The dihedral angle between the C1—C6 and C10—C15 benzene rings is 89.2 (2)°, indicating they are nearly perpendicular to each other. All the bond lengths are comparable to those observed in related structures (Cao, 2009; Xu *et al.*, 2009; Shafiq *et al.*, 2009) and those we reported previously.

In the crystal structure, molecules are linked through N—H···O hydrogen bonds, to form one-dimensional chains running along the *c* axis (Fig. 2 and Table 1).

### Experimental

The title compound was prepared by the condensation reaction of 4-dimethylaminobenzaldehyde (0.05 mol, 7.5 g) and 2-methoxybenzohydrazide (0.05 mol, 8.3 g) in anhydrous methanol (200 ml) at ambient temperature. Colourless block-shaped single crystals suitable for X-ray structural determination were obtained by slow evaporation of the solution for a period of 13 d.

### Refinement

H atoms were positioned geometrically and constrained to ride on their parent atoms, with C–H distances of 0.93–0.96 Å, and with *U*_iso_(H) = 1.2*U*_eq_(C) and 1.5*U*_eq_(C_methyl_). In the absence of significant anomalous dispersion effects, Friedel pairs were averaged.

### Crystal data

**Table:**

C_17_H_19_N_3_O_2_	*D*_x_ = 1.256 Mg m^−^^3^
*M**_r_* = 297.35	Mo *K*α radiation, λ = 0.71073 Å
Orthorhombic, *P**b**c**a*	Cell parameters from 1881 reflections
*a* = 24.726 (4) Å	θ = 2.5–24.0°
*b* = 15.385 (3) Å	µ = 0.08 mm^−^^1^
*c* = 8.2700 (15) Å	*T* = 298 K
*V* = 3146.0 (10) Å^3^	Block, colourless
*Z* = 8	0.20 × 0.17 × 0.13 mm
*F*(000) = 1264	

### Data collection

**Table:**

Bruker SMART CCD area-detector diffractometer	2096 independent reflections
Radiation source: fine-focus sealed tube	1545 reflections with *I* > 2σ(*I*)
graphite	*R*_int_ = 0.045
ω scans	θ_max_ = 22.7°, θ_min_ = 1.7°
Absorption correction: multi-scan (*SADABS*; Bruker, 2001)	*h* = −26→26
*T*_min_ = 0.983, *T*_max_ = 0.989	*k* = −13→16
12277 measured reflections	*l* = −8→8

### Refinement

**Table:**

Refinement on *F*^2^	Primary atom site location: structure-invariant direct methods
Least-squares matrix: full	Secondary atom site location: difference Fourier map
*R*[*F*^2^ > 2σ(*F*^2^)] = 0.039	Hydrogen site location: inferred from neighbouring sites
*wR*(*F*^2^) = 0.106	H-atom parameters constrained
*S* = 1.04	*w* = 1/[σ^2^(*F*_o_^2^) + (0.0545*P*)^2^ + 0.2933*P*] where *P* = (*F*_o_^2^ + 2*F*_c_^2^)/3
2096 reflections	(Δ/σ)_max_ < 0.001
202 parameters	Δρ_max_ = 0.11 e Å^−^^3^
0 restraints	Δρ_min_ = −0.15 e Å^−^^3^

### Special details

**Table:**

Geometry. All e.s.d.'s (except the e.s.d. in the dihedral angle between two l.s. planes) are estimated using the full covariance matrix. The cell e.s.d.'s are taken into account individually in the estimation of e.s.d.'s in distances, angles and torsion angles; correlations between e.s.d.'s in cell parameters are only used when they are defined by crystal symmetry. An approximate (isotropic) treatment of cell e.s.d.'s is used for estimating e.s.d.'s involving l.s. planes.
Refinement. Refinement of *F*^2^ against ALL reflections. The weighted *R*-factor *wR* and goodness of fit *S* are based on *F*^2^, conventional *R*-factors *R* are based on *F*, with *F* set to zero for negative *F*^2^. The threshold expression of *F*^2^ > σ(*F*^2^) is used only for calculating *R*-factors(gt) *etc*. and is not relevant to the choice of reflections for refinement. *R*-factors based on *F*^2^ are statistically about twice as large as those based on *F*, and *R*- factors based on ALL data will be even larger.

### Fractional atomic coordinates and isotropic or equivalent isotropic displacement parameters (Å^2^)

**Table:**

	*x*	*y*	*z*	*U*_iso_*/*U*_eq_	
O1	0.22678 (6)	0.40089 (10)	−0.07729 (18)	0.0681 (5)	
O2	0.22007 (6)	0.33552 (10)	0.36694 (18)	0.0635 (5)	
N1	0.18746 (6)	0.26778 (11)	0.1458 (2)	0.0497 (5)	
H1	0.1951	0.2438	0.0491	0.060*	
N2	0.14034 (6)	0.24198 (11)	0.2235 (2)	0.0485 (5)	
N3	−0.07797 (7)	0.02776 (13)	0.3833 (2)	0.0649 (5)	
C1	0.27573 (9)	0.37736 (13)	−0.0152 (3)	0.0529 (6)	
C2	0.27502 (8)	0.33335 (12)	0.1323 (2)	0.0456 (5)	
C3	0.32355 (9)	0.30874 (14)	0.2011 (3)	0.0611 (6)	
H3	0.3234	0.2808	0.3009	0.073*	
C4	0.37239 (10)	0.32468 (17)	0.1251 (4)	0.0754 (8)	
H4	0.4047	0.3059	0.1707	0.090*	
C5	0.37218 (11)	0.36868 (17)	−0.0186 (4)	0.0784 (8)	
H5	0.4048	0.3805	−0.0699	0.094*	
C6	0.32491 (11)	0.39565 (17)	−0.0884 (3)	0.0700 (7)	
H6	0.3258	0.4264	−0.1852	0.084*	
C7	0.22616 (11)	0.44770 (17)	−0.2266 (3)	0.0836 (8)	
H7A	0.2432	0.4134	−0.3092	0.125*	
H7B	0.2454	0.5014	−0.2138	0.125*	
H7C	0.1894	0.4597	−0.2570	0.125*	
C8	0.22464 (8)	0.31366 (12)	0.2249 (3)	0.0454 (5)	
C9	0.11475 (8)	0.18206 (15)	0.1511 (3)	0.0511 (6)	
H9	0.1286	0.1607	0.0545	0.061*	
C10	0.06471 (8)	0.14545 (13)	0.2125 (2)	0.0477 (5)	
C11	0.04784 (8)	0.06446 (15)	0.1597 (3)	0.0575 (6)	
H11	0.0683	0.0359	0.0817	0.069*	
C12	0.00212 (8)	0.02449 (15)	0.2177 (3)	0.0595 (6)	
H12	−0.0070	−0.0307	0.1808	0.071*	
C13	−0.03087 (8)	0.06541 (14)	0.3311 (2)	0.0505 (6)	
C14	−0.01414 (8)	0.14694 (15)	0.3850 (3)	0.0578 (6)	
H14	−0.0349	0.1759	0.4618	0.069*	
C15	0.03229 (8)	0.18573 (14)	0.3275 (3)	0.0559 (6)	
H15	0.0421	0.2402	0.3666	0.067*	
C16	−0.09234 (10)	−0.05974 (17)	0.3378 (3)	0.0803 (8)	
H16A	−0.0627	−0.0981	0.3608	0.120*	
H16B	−0.1004	−0.0616	0.2243	0.120*	
H16C	−0.1235	−0.0778	0.3981	0.120*	
C17	−0.11115 (9)	0.07019 (18)	0.5026 (3)	0.0823 (8)	
H17A	−0.1181	0.1290	0.4696	0.124*	
H17B	−0.0927	0.0703	0.6048	0.124*	
H17C	−0.1448	0.0396	0.5131	0.124*	

### Atomic displacement parameters (Å^2^)

**Table:**

	*U*^11^	*U*^22^	*U*^33^	*U*^12^	*U*^13^	*U*^23^
O1	0.0796 (12)	0.0712 (11)	0.0536 (10)	−0.0085 (9)	0.0061 (8)	0.0234 (8)
O2	0.0784 (11)	0.0708 (10)	0.0414 (9)	−0.0189 (8)	0.0174 (8)	−0.0065 (8)
N1	0.0541 (10)	0.0541 (11)	0.0409 (10)	−0.0082 (9)	0.0165 (8)	−0.0036 (9)
N2	0.0496 (10)	0.0499 (11)	0.0459 (11)	−0.0043 (9)	0.0139 (8)	0.0023 (9)
N3	0.0521 (12)	0.0772 (14)	0.0653 (13)	−0.0155 (10)	0.0051 (10)	0.0058 (11)
C1	0.0655 (15)	0.0453 (13)	0.0479 (14)	−0.0098 (11)	0.0155 (12)	−0.0028 (11)
C2	0.0550 (14)	0.0359 (11)	0.0458 (13)	−0.0035 (10)	0.0138 (10)	−0.0035 (10)
C3	0.0631 (16)	0.0563 (14)	0.0639 (15)	−0.0020 (12)	0.0095 (13)	−0.0019 (12)
C4	0.0549 (15)	0.0767 (18)	0.095 (2)	−0.0044 (13)	0.0095 (14)	−0.0162 (17)
C5	0.0687 (18)	0.0812 (19)	0.085 (2)	−0.0273 (15)	0.0342 (16)	−0.0231 (17)
C6	0.0837 (19)	0.0681 (16)	0.0581 (16)	−0.0265 (14)	0.0266 (15)	−0.0032 (13)
C7	0.125 (2)	0.0708 (17)	0.0551 (16)	−0.0141 (16)	0.0011 (15)	0.0205 (14)
C8	0.0575 (13)	0.0362 (11)	0.0423 (13)	−0.0011 (10)	0.0121 (11)	0.0031 (10)
C9	0.0520 (13)	0.0587 (14)	0.0425 (13)	−0.0002 (11)	0.0080 (10)	−0.0007 (11)
C10	0.0466 (12)	0.0553 (14)	0.0413 (12)	0.0003 (10)	0.0009 (10)	−0.0011 (11)
C11	0.0540 (14)	0.0641 (15)	0.0545 (14)	−0.0009 (12)	0.0057 (11)	−0.0100 (12)
C12	0.0548 (14)	0.0567 (14)	0.0671 (16)	−0.0072 (12)	−0.0045 (12)	−0.0058 (12)
C13	0.0440 (12)	0.0620 (15)	0.0455 (13)	−0.0029 (11)	−0.0064 (10)	0.0062 (12)
C14	0.0497 (13)	0.0691 (16)	0.0545 (14)	−0.0016 (12)	0.0086 (11)	−0.0074 (12)
C15	0.0547 (13)	0.0534 (13)	0.0597 (14)	−0.0066 (11)	0.0042 (11)	−0.0074 (12)
C16	0.0687 (16)	0.0782 (19)	0.0941 (19)	−0.0233 (14)	−0.0054 (14)	0.0165 (16)
C17	0.0606 (15)	0.114 (2)	0.0728 (17)	−0.0136 (15)	0.0145 (14)	0.0077 (17)

### Geometric parameters (Å, °)

**Table:**

O1—C1	1.363 (2)	C7—H7A	0.9600
O1—C7	1.429 (3)	C7—H7B	0.9600
O2—C8	1.227 (2)	C7—H7C	0.9600
N1—C8	1.331 (2)	C9—C10	1.451 (3)
N1—N2	1.389 (2)	C9—H9	0.9300
N1—H1	0.9002	C10—C11	1.385 (3)
N2—C9	1.269 (2)	C10—C15	1.390 (3)
N3—C13	1.370 (3)	C11—C12	1.373 (3)
N3—C17	1.440 (3)	C11—H11	0.9300
N3—C16	1.442 (3)	C12—C13	1.393 (3)
C1—C6	1.387 (3)	C12—H12	0.9300
C1—C2	1.396 (3)	C13—C14	1.394 (3)
C2—C3	1.381 (3)	C14—C15	1.379 (3)
C2—C8	1.493 (3)	C14—H14	0.9300
C3—C4	1.384 (3)	C15—H15	0.9300
C3—H3	0.9300	C16—H16A	0.9600
C4—C5	1.367 (4)	C16—H16B	0.9600
C4—H4	0.9300	C16—H16C	0.9600
C5—C6	1.368 (4)	C17—H17A	0.9600
C5—H5	0.9300	C17—H17B	0.9600
C6—H6	0.9300	C17—H17C	0.9600
			
C1—O1—C7	117.94 (17)	N1—C8—C2	115.55 (18)
C8—N1—N2	120.22 (16)	N2—C9—C10	122.83 (19)
C8—N1—H1	120.6	N2—C9—H9	118.6
N2—N1—H1	118.0	C10—C9—H9	118.6
C9—N2—N1	114.06 (16)	C11—C10—C15	116.31 (19)
C13—N3—C17	120.6 (2)	C11—C10—C9	119.73 (19)
C13—N3—C16	121.5 (2)	C15—C10—C9	123.93 (19)
C17—N3—C16	117.47 (19)	C12—C11—C10	122.7 (2)
O1—C1—C6	124.1 (2)	C12—C11—H11	118.6
O1—C1—C2	116.54 (18)	C10—C11—H11	118.6
C6—C1—C2	119.4 (2)	C11—C12—C13	121.0 (2)
C3—C2—C1	118.84 (19)	C11—C12—H12	119.5
C3—C2—C8	117.25 (19)	C13—C12—H12	119.5
C1—C2—C8	123.87 (19)	N3—C13—C12	121.2 (2)
C2—C3—C4	121.5 (2)	N3—C13—C14	122.1 (2)
C2—C3—H3	119.2	C12—C13—C14	116.62 (19)
C4—C3—H3	119.2	C15—C14—C13	121.8 (2)
C5—C4—C3	118.6 (2)	C15—C14—H14	119.1
C5—C4—H4	120.7	C13—C14—H14	119.1
C3—C4—H4	120.7	C14—C15—C10	121.6 (2)
C4—C5—C6	121.3 (2)	C14—C15—H15	119.2
C4—C5—H5	119.3	C10—C15—H15	119.2
C6—C5—H5	119.3	N3—C16—H16A	109.5
C5—C6—C1	120.2 (2)	N3—C16—H16B	109.5
C5—C6—H6	119.9	H16A—C16—H16B	109.5
C1—C6—H6	119.9	N3—C16—H16C	109.5
O1—C7—H7A	109.5	H16A—C16—H16C	109.5
O1—C7—H7B	109.5	H16B—C16—H16C	109.5
H7A—C7—H7B	109.5	N3—C17—H17A	109.5
O1—C7—H7C	109.5	N3—C17—H17B	109.5
H7A—C7—H7C	109.5	H17A—C17—H17B	109.5
H7B—C7—H7C	109.5	N3—C17—H17C	109.5
O2—C8—N1	123.55 (18)	H17A—C17—H17C	109.5
O2—C8—C2	120.81 (19)	H17B—C17—H17C	109.5

### Hydrogen-bond geometry (Å, °)

**Table:**

*D*—H···*A*	*D*—H	H···*A*	*D*···*A*	*D*—H···*A*
N1—H1···O2^i^	0.90	2.03	2.914 (2)	165

Symmetry codes: (i) *x*, −*y*+1/2, *z*−1/2.
